# Factors supporting research capacity development in an English local authority: a qualitative description and comparison with proposed mechanisms

**DOI:** 10.1186/s12961-026-01469-2

**Published:** 2026-02-28

**Authors:** Shannon M. Kennedy, Annette Haywood, Susan Hampshaw, Jo Cooke

**Affiliations:** 1 Sheffield Centre for Health and Related Research, Division of Population Health, School of Medicine and Population Health, University of Sheffield, Sheffield, UK; 2 Yorkshire and Humber School of Public Health, NHS England, Leeds, UK; 3 Division of Population Health, School of Medicine and Population Health, University of Sheffield, Sheffield, UK; 4 City of Doncaster Council, Doncaster, UK; 5 School of Allied Health Profession, Nursing and Midwifery, University of Sheffield, Sheffield, UK

**Keywords:** Co-production, Determinants of health, Framework analysis, Leadership, Local authorities, Public value, Research capacity development, Research culture, Research skills

## Abstract

**Background:**

Research capacity development (RCD) involves resourced and sustained multi-faceted skill-building to enable high-quality, useful research alongside social change. Local authorities (LAs) have substantial potential to enhance health and wellbeing through research engagement given their role shaping determinants of health, access to rich data and interest in “what works”. This paper presents a qualitative reanalysis of research interviews undertaken in an NIHR funded Local Authority Research Systems (LARS) project in Doncaster Council, an upper-tier LA in England. It considers RCD mechanisms developed in health and care and public service literature to extend theoretical insights into RCD in English local government.

**Methods:**

Initial project sampling was purposive, using a snowball approach. Interviewee work areas spanned adult and young people’s services. The interview schedule drew on mechanisms identified in published literature. Secondary thematic framework analysis of the transcripts of the 15 study interviews also used a deductive frame of mechanisms from published literature. Secondary analysis included discussion and review among the authors to refine themes and analysis.

**Results:**

Interviewees identified mechanistic and cultural determinants of research capacity in this LA. Key themes were collaboration; making a difference; indications of expectations and importance of research; relationship between research, practice and policy; resource and capacity; and research as everyone’s business. Our findings support the relevance of previously published RCD mechanisms to the LA context. Some mechanisms resonated more than others: “exceeding the sum of parts”, “coproduction” and “feeling you are making a difference”. The latter of these mechanisms resonated strongly.

**Conclusions:**

RCD mechanisms can stimulate joint work, promote synergy and unlock potential. Research partnerships should focus on local impact for communities and balance between scientific rigour and actionable research. Success relies on dialogue and co-production across research, health and care, and departments within LAs to address the wider determinants of health. This includes setting joint priorities, brave leadership and power-sharing between stakeholders. LAs can take steps including making research core business, ensuring protected time and experiential learning, and community involvement in research. Lessons from this study may offer transferable insights that inform RCD in comparable local government settings.

## Study background

### Research capacity development (RCD)

RCD has at times been characterized by complex and sometimes ambiguous conceptual framing [1, 2]. More recently, it has benefitted from concept analysis describing RCD as “a funded, dynamic intervention operationalized through a range of foci and levels to augment ability to carry out research or achieve objectives in the field of research over the long-term, with aspects of social change as an ultimate outcome” [3]. It has strong associations with a body of literature broadly situated in healthcare systems, and in the United Kingdom, this has meant it has been understood as a core National Health Service (NHS) capability [3–5]. The movement of United Kingdom public health responsibilities out of the NHS and into local government in 2013 highlights the importance of considering RCD within this setting, where so much of health is created, and therefore where relevant research might well be situated [6].

Related to concepts such as knowledge mobilization and knowledge translation [7], RCD describes a particular, researched-focused facet of the broader concept of capacity development. Instead of being limited to a hierarchical, one-way flow, RCD seeks to develop research capacity at all parts and levels of a system, taking an inclusive view of fostering the ability to engage with research [8]. While the concept of developing (or building) capacity has faced criticism of its conceptual ambiguity and breadth [9, 10], RCD is sometimes conflated with research capacity building (RCB). However, some authors make the distinction between RCB – where “building” implies establishing capacity that does not already exist – and RCD – where “development” can include both initial creation and improvement or strengthening of capacity that might already be present [5]. The present authors agree with this distinction, using RCD through this study to recognize that research capacity was present to some degree in the study site.

The importance of RCD lies in its potential to drive improved interventions and effectiveness in both healthcare and public health practice, and so ultimately to deliver better outcomes for the public. Developing research capacity within a system can help foster actionable insight, not only increasing knowledge but also helping ensure this influences practice [7]. RCD is essential as a foundation of a research culture underpinning positive transformation in the near and longer term [5]. While the NHS Constitution commits the United Kingdom health service to “conduct and use of research to improve the current and future health and care of the population” [11], there should be an equal imperative to develop the capacity for such a research culture in English local authorities given their central role in shaping the health of the public and preventing illness [6].

### English local authorities

The United Kingdom, and England in particular, has a distinctly centralized system of government relative to comparable countries [12]. The main body of English local government commissioning and delivering local services such as public health is the upper-tier authority (not all areas have any additional lower tiers) [12, 13]. The terms “local council”, “local authority” and “local government” generally refer to this tier, particularly in respect to public health [6, 14]. This study focused on this upper tier in the form of a unitary council.

These local councils provide and commission services and shape policy, which has an impact on people’s health and wellbeing. English local councils have held responsibility for the public health of their populations since 2013, a move which built on the powers over economic, environmental and social wellbeing conferred to local authorities by the Local Government Act 2000 [12, 13]. Beyond public health itself, English local authorities are responsible for housing, the environment, education and green spaces, and if these services are informed and influenced by evidence, they have the potential to affect upstream determinants of health and reduce inequalities [15–17]. English local councils therefore have great potential to improve people’s lives through undertaking and using research [18].

English local authorities also collect and interpret a diverse body of real-world data, ranging from details on infant feeding to tobacco control [13]. This insight is relevant to health and has a great potential to test out what works in a timely way, which can support evidence-based transformation of services in the health and care systems [18]. However, a recent study has highlighted difficulties around engagement and use of research within local government and describes a lack of understanding of what research entails, including a limited knowledge of how to engage in research [19]. Two reviews provide similar messages. The first [20] examined research culture and evidence in relation to population health improvement in local authority spaces, principally through Local Authority Champions of Research (LACoRs). It concluded that there was indeed an appetite for conducting and developing research in local government but found there were no shared views about what counts as evidence and that multiple cultures of evidence and evidence use co-exist in the same organization. The second review [21] highlighted both an inconsistent understanding of, and lack of meaningful literature addressing, local authority research systems.

This mismatch between the potential and reality has been recognized by the UK National Institute for Health and Care Research (NIHR) which developed an approach to developing research infrastructure in local government in the form of Health Determinants Research Collaborations (HDRCs). The aim of HDRCs is to “enable local authorities to become more research-active, using evidence to inform their decision making by undertaking research and evaluation relating to their activities, including synthesising and mobilising existing evidence” [22]. HDRCs have an ambition to be centres of research excellence, with a strong emphasis on developing research capacity in local government *and* the academic workforce so they can collaborate together in ways that are appropriate and meaningful to local government. This crucial funding and the requirement of HDRCs to be centred in local authorities are important elements for programmes of relevant, high-quality research to inform policy and practice for real-world impact. Within roughly the first year after the initial group of HDRCs were established, NIHR had funded 30 of the collaborations. Their partnership nature means that not only do HDRCs foster collaboration between LA and academia, for example through embedded researchers, but some HDRCs are also collaborating with one another to become more than the sum of their parts. As HDRCs share and publish their findings over coming years, the relatively limited evidence base on RCD in local authorities can be expected to grow considerably and beneficially [22].

### RCD and local authorities

RCD is embedded in, and influenced by, context and social meaning. The dentition of evidence is itself contested, and standpoints of institution and discipline or subject also influence what is valued as evidence [23–25]. Local authorities may define evidence differently as compared with the NHS for example, or vary in value ascribed to evidence sources and their appropriateness for enabling impact [14, 26]. Public health decisions and policy are infrequently based on research evidence as compared with sources of local evidence [14, 27, 28] and, in some contexts such as public policy, the gold standard value of RCD evidence itself is contested [29]. Furthermore, evidence in the context of health policy and delivery may encompass a duality of both active processes and the more familiar body of evidence [30].

Therefore, when undertaking RCD within local government, we should be cognisant about developing skills and an environment suitable for undertaking appropriate research. But what counts as appropriate research? Many have argued that a co-production approach that focuses on local impact is required, in contrast with the emphasis on generalizable findings in mainstream health research [17, 31]. While engagement such as public and patient involvement (PPI) has a role in academic and clinical research, it is arguably more central to a democratically accountable local government context [17, 32, 33]. Given that existing health and care research systems are not designed to meet local authority requirements, the question arises as to whether proposed mechanisms and programme theories of what works for RCD derived from non-local authority contexts are amenable to council settings and to what degree? This is the key interest of the present study which was funded by the NIHR in a small grant, as part of a funding round addressing local authority research systems. This paper presents a secondary analysis of the data through the lens of RCD mechanisms developed in a 2018 realist review [8] of the health and care literature to explore and extend the theoretical insight into RCD in local government.

## Methods

The aim of this present study was to undertake a secondary analysis of data drawn from research interviews undertaken in an earlier NIHR funded Local Authority Research Systems (LARS) project in one authority in the north of England. That earlier project aimed to explore the appetite of senior officers and elected members to increase the local authority’s organizational capacity to undertake research. A researcher attached to Doncaster Council lead the data collection through semi-structured interviews with local government officers and elected members. They also undertook the data analysis. The secondary analysis forming the basis of this present study aimed to explore these earlier data through the lens of overarching programme theory mechanisms identified by Cooke and colleagues’ 2018 realist review (Table 1). The purpose for undertaking this secondary analysis was to explore the RCD mechanisms in terms of relevance and applicability in this context and to build a greater theoretical insight into what might be appropriate for action.

**Table 1:** Cooke and colleagues’ proposed RCD mechanisms [8]

Overarching programme theory	Research capacity will be affected if…
PT 1: Exceeding the sum of the parts	Individuals/organizations/networks realize a contribution that they are unlikely or less likely to achieve in isolation (e.g. grant writing groups, communities of practice, doctoral training networks)
PT 2: Learning by doing	Individuals/organizations prototype or practise activities required for subsequent full engagement, and sequentially learn through cycles of reflection
PT 3: Liberating the talents	Individuals/organizations release the dormant potential of their skills and experience
PT 4: Releasing resources	Resources provided to overcome individual/organizational inhibition and act as a focus for activity, and information is freely shared about these opportunities
PT 5: Co-producing knowledge	Individuals/organizations share ideas and knowledge development through networks and partnerships
PT 6 (s): Feeling that you are making a difference	Individuals/organizations perceive that research has an impact on health/wealth/knowledge creation/tackling inequalities
PT 7 (s): Modelling positive behaviours	Individuals observe the positive impact of involvement in research by others in the organization
PT 8 (s): Signalling importance and making research core business’	Individuals perceive that involvement in research is a valid activity in relation to competing priorities within the organization

The sampling for the primary data collection in the initial LARS project was purposive to ensure representation across different role types and areas of work across the council, with selection criteria based on working as a senior (specialist or management-level) officer or serving as an elected member within Doncaster Council, and self-reported interest and ability to take part in an interview about research within the local authority. To maximize professional networks within the council, sampling was on the basis of a snowball approach, initiated through nominations from Heads of Service within the council and email invitations to identified and potentially suitable council officers and elected members who in turn recommended further participants to interview until saturation was reached.

Interviews were recorded using Microsoft Teams, and transcribed by the original NIHR study team. This resulted in 15 transcribed semi-structured interviews with 13 senior local government officers from four council Directorates (Adults, Health and Well Being; Corporate Resources; Economic and Environment; and Learning and Opportunities – Children and Young People) and two elected members. The interviewees included heads of directorates (2), directors and assistant directors (4), managers (1), strategic leads (1), social workers (1), educational psychologists (1), public health professionals (3) and locally elected politicians (councillors) (2). Work areas included strategic commissioning, communities and localities, and policy. These spanned adult and young people’s services, including health and social care, and education. The interview schedule was based on the mechanisms identified in the 2018 realist synthesis [8].

The secondary analysis involved thematic analysis of the previous NIHR study’s interview transcripts using a framework approach. This method allowed for the recognition of shared features and divergences in the interview data. Framework methodology is particularly well-suited to applied qualitative research, as in the case of our study [34]. It is an approach that begins from a deductive base with known questions or themes, but also allows for emergent, inductive thematic codes to be identified through the process of data analysis [35]. This strong deductive base to establish the frame also offered the opportunity for emergent themes from the data, making it an appropriate method for our analysis, given our aim to consider our interview data in reference to the RCD mechanisms [8] previously characterized. The particular English local authority context of our study was expected to yield additional inductive themes from interviewee insights, or contextualization. That is because framework methodology includes displaying data extracts from all codes in a frame or matrix so even data that diverges from the overarching theme trend is included in analysis [35].

Comparison of interview data with proposed theories and mechanisms in the 2018 synthesis [8] grounded and shaped the discussion in moving from reporting to interpreting the results of the analysis. That study [8] focused on 36 conceptual and theoretical papers, but the English local government context did not feature among those. Our data analysis was therefore intended to identify whether and how our local authority interview data converged and/or diverged when framed by the identified RCD mechanisms. The focus on these identified RCD mechanisms underpinned our investigation because they go beyond describing RCD to identifying the mechanisms serving as a foundation to RCD. This insight was relevant to the council from which the interviews were drawn as they wished to find practicable means of enhancing their research culture and engaging in RCD. By focusing on the mechanisms, we wished to elucidate the foundations of RCD activity to understand mechanistically the themes of what drives RCD. Using the 2018 mechanisms [8] as the frame for our secondary analysis in this study, we assessed the potential relevance of those mechanisms to our study’s local authority, which embarked on new plans to act on RCD. The member of the study team undertaking this secondary transcript analysis shared initial themes and subthemes with members of the earlier NIHR primary data collection team for their insight to inform data analysis.

## Results

Our results comprised interviewees' views across activities, infrastructure and a supportive research culture. They drew on both past and ongoing experiences, offering ideas for future work on keystones, barriers and enablers of doing and using more and better research in their context. Informants shared a positive attitude to research and perceived the same disposition, particularly from senior leaders. This sense that research was important was seen as the basis for promoting research expectations and supporting RCD activities. Identified challenges concerned how to put research appetite into practice, join up work taking place, and access funding or other resource – of moving from abstract to practical investment and support. These were considered precursors both to utilize existing research capacity effectively and develop it through training and experience, embedding research in all the council does.

The themes identified through the secondary analysis of the data map out against the mechanisms given in Table 2. These themes will be described by mechanism and provide evidence to assess how amenable each are for the local authority context and what issues may entail to trigger such mechanisms in local government RCD practice.

**Table 2:** How our study themes map against the RCD mechanisms

Mechanism for RCD [8]	Relevant theme from our secondary analysis
PT 1: Exceeding the sum of the parts	Collaboration among professionals Learning from others Connections, networks, relationships
PT 2: Learning by doing	Training
PT 3: Liberating the talents	Untapped talent and resource Research loci
PT 4: Releasing resources	Allocating and using resources Competing priorities
PT 5: Co-producing knowledge	Working for and with citizens Interconnections between research, policy and practice
PT 6 (s): Feeling that you are making a difference	Making an Impact Responsive and democratic
PT 7 (s): Modelling positive behaviours	Role models and celebration through events
PT 8 (s): Signalling importance and making research core business	Expectations of being research-led Embedding research in all we do

### Exceeding the sum of the parts

Three themes from the secondary analysis linked to this mechanism, which Cooke and colleagues [8] explain can be enacted when “individuals/organisations/networks realise a contribution that they are unlikely or less likely to achieve in isolation (e.g., grant writing groups, communities of practice, doctoral training networks)”. In our study, it emerged as themes related to collaboration among professionals, learning from others, and the demonstrable and perceived importance of connections, networks and relationships.

#### Collaborations amongst professionals

Experience within the council included working with internal colleagues and across external organizations and individuals to do research. External collaborators included academic partners, the NHS, charities, national bodies and government. This enabled participation in larger-scale research and sharing research capacity. Academic researchers were perceived to provide rigour to research. Collaborations were enabled through pursuing mutual interest.

*If you go to a researcher or somebody who’s sort of interested in research who are in academia. As long as it’s something that remotely sort of falls into their area of interest usually, they are really keen to get involved.* (I2)

Collaboration with the public to guide research or undertake research was more prominently seen as highly relevant and imperative. Indeed, a whole-system approach was understood to necessarily include the public. Public–officer collaboration is explored in more detail under the co-production principle.

#### Learning from others

Learning from good practice elsewhere was seen as important and responsive to address emergent challenges, or to bring research findings to bear on their own work.

*We have been looking at research that has been done [elsewhere] and looking at how we can take the best bits from that.* (I16)

However, external evidence was not always sufficient to inform local developments.

*For the longest time we have been putting all the best practice guidance into action […] And it feels like we are doing everything we are being asked to do, or that we should be doing, and yet we haven’t seen any impact at all.* (I2)

#### Connections, networks, relationships

Most interviewees discussed links to people, groups and institutions being instrumental to RCD and its subsequent research utilization. Networks and connections could be enablers to research where present, and barriers where absent.

*It’s the relationships that create the better opportunities.* (I6)

Initial collaborations were often instigated through research projects and training, and such contacts could precipitate longer-term relationships that fostered ongoing joint research activity. Some interviewees, however, recognized that not everyone has these links and building relationships with research experts could be intimidating where officers lacked prior experience.

*My connections with [university] purely comes from me studying there and me knowing people there. So I guess it might be a bit difficult for people if… how do you even start to approach an academic if you’ve got no experience?* (I2)

### Learning by doing

For Cooke and colleagues [8], this mechanism in one where research capacity was developed when “individuals/organisations prototype or practise activities required for subsequent full engagement, and sequentially learn through cycles of reflection”. In our interview data, this was described through a focus on training and developing including through informal or practical means.

#### Training

Respondents highlighted the need for training on research methods, but such training should recognize current strengths. Training was considered useful if it could support the potential to do purposeful and rigorous research.

*I think at an individual level it definitely needs to work on confidence and people’s interests. So, building up those research skills and drawing on the strengths and expertise that people already have.* (I4)

Several respondents felt colleagues would embrace training and hands-on skill or confidence-building in methods and data interpretation and data competence. Respondents highlighted the present lack of research training or development opportunities even when included in personal development plans. There was a general lack of awareness of whether research training or RCD development resource was available, and of what form this might take, such as secondment or external training opportunities.

### Liberating the talents

As described in the 2018 synthesis [8], this mechanism affects RCD when “individuals/organisations release the dormant potential of their skills and experience”. We found our interviewees considered this theme in terms of untapped talent and resource and the locus or loci of research within the system.

A common theme amongst the interviews was that talent and potential to contribute to research was there but not liberated.

#### Untapped talent and resource

Many respondents recognized the great potential for research to be undertaken in a local authority setting, but that this was an untapped resource. An interviewee noted the richness of research opportunities offered in the area.

*I find it amazing we’ve got all these contacts, social work, Family Hubs, that are rich places to do research and we’ve got it all at our fingertips.* (I8)

*We really have some knowledgeable skilled staff and I think that would be good to use their knowledge and their skills.* (I11)

Others recognized that they could apply skills and experience to support more research than their present role allowed, while others felt an inability to be research-active led to people being deskilled.

*I suppose sometimes I just feel a bit rusty, and a bit old.* (I3)

#### Research loci

There was also a perception by some that only certain teams, such as in public health, were seen as enclaves of research activity and expertise.

*… I do feel like there’s a perception of who’s an expert in the council and they always go to the same people who they perceive as experts in that area.* (I4)

This compounded the concentration of research opportunities on these colleagues and forestalled the broadening of RCD to others. A few interviewees indicated that research experts in teams held knowledge and information about relevant activity but that this was untapped and unrecognized.

*When people feel like research is the preserve of experts or people distant from them, they don’t have any skin in the game themselves.* (I15)

*‘Oh we’ll leave it to the Adult Social Care, Public Health, because they like that type of thing,’ whereas for me as a council, what we should be doing, our Neighbourhood Teams could be part of research.* (I12)

### Releasing resources

This mechanism is described by Cooke and colleagues [8] as related to when “resources provided to overcome individual/organisational inhibition and act as a focus for activity, and information is freely shared about these opportunities”. Our respondents were particularly focused on the needs and challenges of allocating resources, and the considerable competing priorities they faced around this in the local government context.

#### Allocating and using resources

Interviewees explained that certain management roles had access to discretionary funding that could contribute to research-related costs. However, in the context of competing priorities teams were often required to secure external funding to be research-active leading to missed opportunities for independent evaluation or development of research partnerships.

*We don’t necessarily have funding to wrap around pilot projects or new work that evaluates and builds that evidence base.* (I16)

Time to undertake research was a significant barrier, particularly if partnership work meant influencing the release of time across teams and directorates. Even where funds to conduct research was awarded, protecting the requisite time could be challenging.

*It’s one thing promoting and wanting that [research] to happen and then another way where that actually manifests in people taking up that opportunity and finding time to do it.* (I5)

#### Competing priorities

Others believed certain colleagues felt research was a low priority to be fit into spare capacity, which could hinder RCD. The context of the COVID pandemic also highlighted issues. Unless the emergencies and incidents subsided, undertaking research in the face of such demands would continue to be challenging.

*[The Council] has just spent like 10 years fighting fires. Now we continue to do so.* (I14)

### Co-producing knowledge

For Cooke and colleagues [8], this mechanism meant RCD occurred when “individuals/organisations share ideas and knowledge development through networks and partnerships”. The partnership and relational development elements of this were particularly meaningful for our interviewees, whose responses linked to this theme in regard to working for and with citizens and their considerations of interconnections between research, policy and practice. This was a strong and important theme from the interview data linked to the need to show research to be immediately relevant. Working with citizens and practice teams was seen as integral to this mechanism and highly relevant to the local authority context.

#### Working for and with citizens

Engagement with citizens was seen as important, conceptualizing research as finding out views from populations to shape services. Being led by “user voice” and involving residents in research were enjoyable and motivational aspects of research.

*Getting people involved, to have their say about what they want and how we can then use that research to bring it back into practice.* (I6)

There was also a strong theme that research should involve community embeddedness, incorporating community or peer researchers to promote trust and credibility in their communities. The reciprocity between research and practice was discussed in relation to both the iterative development of interventions within research and service development, and the need for evidence that shapes practice to take account of user or resident voice to ensure that the resulting policies and practices reflect these views. Evidence from practice and citizens was seen as imperative to undertake RCD in local authorities.

#### Interconnections between research, policy and practice

Using and doing research in tandem was seen as a positive cyclical approach where evidence informs policy and practice within and alongside evaluation and could have the potential to spur further action. This further supports the notion of co-production with practitioners and senior officers.

*I guess it will steer us in terms of when new evidence comes to light around certain areas we might want to change tack. So I suppose it informs us and at the same time we can create the knowledge ourselves and be the evidence as well as drawing on what other people have done.* (I2)

### Feeling that you are making a difference

In the 2018 study [8], this mechanism is enacted when “individuals/organisations perceive that research has an impact on health/wealth/knowledge creation/tackling inequalities”. This is a clear means of linking research to the very real aim and objectives of English local government. Our analysis showed a particularly local-authority-focused presentation of this mechanism, where respondents described the importance of making an impact for local people while noting the importance of doing this in ways that were responsive and democratic. This again was a highly supported theme from the data but extends the concept of the mechanism. Rather than simply feeling research makes a difference, it was important to *demonstrate* that research makes an immediate difference.

#### Making an impact

A common driver of doing and using research was the chance to identify what works and deliver outcomes and improvements for local people. This was seen as a strong motivation to support wider engagement and increase RCD.

*[If]… people can see the benefit of it, I suppose it increases the buy-in.* (I1)

Research was considered amenable for tackling both new and longer-term issues that have persisted in the area despite the application of best-practice guidelines. This conception merged personal motivations to make a difference to people, and organizational objectives to optimize efficiency.

*We have to do the trials and the testing and thinking of new ways to do things. We want to see impact, and we want to see outcomes, and if we are not getting that, why aren’t we and what do we need to do differently?* (I2)

Responses indicated the importance of both skilled internal and independent evaluation, and developing this capacity into programmes of work where it was lacking currently. This highlights a tension between seeing a difference but understanding it takes time, particularly in the political environment of local government. Some also described challenges of ensuring adequate duration of data collection and follow-up to see impact.

*It’s really hard sometimes in our work to be able to demonstrate an impact straight away[…] [it is] sometimes really, really hard to say, ‘has that made a difference?’ because it could take a year, 2 years… I think one of the things that we are not good at, and I think one of the things that we need to think about, is that longitudinal [perspective] – how do we go back in a year’s time and say, ‘did that work?’ And how can we go back and ask people that… ?* (I7)

#### Responsive and democratic

Research was often seen to help understand population needs and interests and was seen to have the potential to support accountability in the use of the local authority’s powers and funds, and to underscore the legitimacy and responsiveness of decisions. A particular feature of responses – prominent in but not limited to responses from elected members – reflected the council’s public duties of responsiveness and democratic accountability. Some also described the onus on councils to follow rules and meet duties, all within consideration of election cycles and issues of electability to councillors, which could have implications for programmes of research regarding funding, timescales and topics of interest. In addition, a council-wide compassionate ethos was seen as a driver of internal research activity.

*If we were wanting to be a compassionate borough, we are wanting to really understand the voice of, and the needs of, and the experiences of our communities and people within there, then I think we need to do it ourselves.* (I9)

### Modelling positive behaviours

This RCD mechanism relies on activity where “individuals observe the positive impact of involvement in research by others in the organisation” [8]. For our respondents, this meant both the presence of research role models who could serve as touch-points or catalysts for more RCD, and of the celebration and showcasing of research and its use.

#### Role models and celebration through events

Modelling positive behaviours was seen in two ways: firstly, focusing on individual research champions and role models, and secondly, in relation to championing events and activities to show how research can make a difference. Both were seen to promote a research culture and wider engagement with research and its practice.

The idea of having role models who are research active and encourage others to get involved in research was seen as an important potential to support RCD in the council. Many identified the value of both internal or external role models for fostering RCD who could champion and mentor others to do and use research. Some respondents identified individuals who already served in this capacity.

Showcase events were seen as important to celebrate success and demonstrate how research is done and how it is used. For example, a research week had been held and respondents expressed pride in this.

*I think it could be something that people could be quite proud of. So, like, a day where they could present what they’ve found, and people could watch them speak about it…* (I4)

*A research week is really, really important […] it kind of sets out… this is, this is something that we’re about as a group of businesses, agencies… that it’s really important to take stock and hear from people doing some really interesting, fantastic stuff.* (I8)

### Signalling importance and making research core business’

In their earlier study, Cooke and colleagues [8] explained that RCD is impacted when “individuals perceive that involvement in research is a valid activity in relation to competing priorities within the organisation”. In our results, this meant the organization and its leaders stating their expectations of the council and its work being research-led. The respondents felt this was linked to the necessity of embedding research in everything the council did, so it was an integral part of systems and processes.

#### Expectations of being research-led

Many interviewees noted a clear appetite for research activity in the council whilst also noting a disparate passion for research between managers and frontline colleagues. Differences across teams and directorates was also recognized, reflecting multiple cultures coexisting in the council. While most felt that senior leaders valued research, some questioned the levels of practical research support they could provide.

*I think there’s an appetite. I don’t think we know how, and I don’t think we’ve encouraged it yet. So I think why this would be really useful. We talk about it and we say, ‘we want to be research-led’.* (I9)

Some questioned whether enthusiasm and awareness filtered to all areas of the council. Some recognized uneven research expectations and a variation across roles and tasks that represented good practice. It was postulated that some teams worked in a “factory culture” that encouraged task-focused rather than reflective working, which tended to lead to continuing with current practice or policy.

#### Embedding research in all we do

Most interviewees discussed the need to proactively incorporate research into all council business rather than remaining the preserve of its purported “home” in certain teams that were seen as a natural or expected place for research activity, for example the public health team. Such ideas included making research part of the induction when people join the local authority, but also more about how everyday decisions are made. Several interviewees proposed embedding research expectations in council processes and approval protocols, suggesting that research-based proposals should be expected in reports and recommendations to senior leaders and the Cabinet.

*I’d like to sit in Executive Board […] that whenever we suggest something that our first thought is, ‘but what’s the research behind this […] And then understand the impacts of that. Change that mentality really of just making decisions because we know best.* (I12)

*Senior Leadership Team to place it central to our decision making and to be given the resource and green light to do this.* (I16)

Embedding research into everyday practice involved leadership style but also the ability to release resources and permissions.

*I think it’s a, it’s a top-down message in terms of that permission and then I think it’s a bottom-up message or action in terms of having time out and with the blessing of your line manager.* (I5)

## Summary

This study’s results identified the importance of both practical actions or infrastructure and symbolic or cultural conditions to develop research capacity. These relate to the five activity-based and three symbolic programme theories from the 2018 synthesis [8]. The activities and infrastructure that interviewees raised in our data were comparable to the five activity-based programme theories in the 2018 synthesis, and support similar symbolic mechanisms for RCD as those three in the same study [8]. The overall agreement between the 2018 synthesis [8] and themes emergent from our study coalesced around some key points from council informants regarding what was important for driving (or, by absence, inhibiting) their ability to use and do better research, the development or improvement of which would support RCD.

They noted the need to address this council’s gap in the development and maintenance of necessary research skills and confidence, achieved by funding training as well as sharing and creating opportunities for practical research experience equitably. They also expressed a strong motivation for research relevant to their duties and the people they served, including local government considerations of democratic accountability and community responsiveness. Interviewees saw a mechanism in having research situated close to policy and practice both in terms of direct utility and being driven by and contextualized by the reality of the place. The ability of collaborations, networks and relationships to enhance RCD (and hinder where it was lacking) was a prevalent interview theme. Maximizing impact through sharing knowledge into and out of their own council settings in terms of publication access and modelling or showcasing research activity were noted. Interviewees discussed ensuring continuity and sustainability of research and RCD activities, avoiding examples of work or interest being lost or falling by the wayside. Finally, enhancing RCD through appropriate infrastructure and systems was expressed by several informants, such as expert process guidance or online information repositories about research activity.

## Discussion

For the most part, our findings support the relevance of the RCD mechanisms to the local government context. Some mechanisms resonated more loudly than others. Those that were strongly supported and which showed evidence of current application include: “exceeding the sum of parts”, “co-production” and “feeling you are making a difference”. The latter of these mechanisms showed the most resonance among respondents, illustrating an appetite for RCD to enhance the common good.

An important message for RCD and supporting a research culture in local government was that research should aim to have a positive impact on local people and services. Joint working and co-production were seen as the means to the end in making the difference. Our findings highlight a subtle but important difference to how this was characterized by Cooke and colleagues. [8]. For local government, the mechanism should be altered to *showing* you make a difference *locally*. The desire for research to make a local impact has been recognized by others [36–38]. This uncovers differences in types of evidence valued in local government compared with mainstream health research and suggests a different hierarchy in this context suggesting actionable evidence is valued more than producing generalizable findings. It also highlights issues and tensions that we might expect to encounter in academic partnerships working with local government. In order to work in a synergistic manner a balance needs to be found between relevance and scientific rigour [20, 36] and setting mutually beneficial agendas with an eye on impact from the outset [39, 40]. This then also informs how the “more than the sum” mechanism might operate. In this way, RCD can be seen as a means for local authorities to increase the common good through cultivating and using new capabilities to set and reach shared system goals, delivering changes that are meaningful to professionals and the public [41].

Others have highlighted the demands of research–practice partnerships, but emphasize the need to work together using co-productive or co-creative approaches [42, 43]. Ways of power sharing need to be achieved through reflexivity and mutual respect to support win–win or tit-for-tat problem solving [17, 20, 36, 40], and adaptive governance structures that are inclusive and support continuous conversations [17, 44]. Effective leadership in public service that champions RCD therefore must include an ability to appreciate the differing priorities that can arise among the range of relevant stakeholders and between those working directly with the public and those in strategic senior roles [41].

These conversations should start by discussing assumptions, expectations and knowledge beliefs in order to uncover shared values and objectives [37, 45]. If this is not undertaken, tensions are likely to develop and persist [46]. Steens and colleagues [36] for example, achieved success in local government research projects through developing partnerships that support a continuous balance between practice knowledge, lived experience and scientific knowledge. Partnership structures do not automatically facilitate meaningful and useful projects, but these can be facilitated by knowledge brokers and expectations management [37, 47]. All these approaches focused on local solutions and practical benefits as an outcome of research, as well as possibilities of transferable lessons learned. Our findings highlight the value of working with diverse groups but particularly with citizens and communities. This is necessary as local government needs to be responsive and democratically and locally accountable. Public participation also offers the possibility of increasing trust and satisfaction among a diverse range of voices, as well as supporting bottom-up policy change that is inclusive and sustainable [48]. There is also an increasing top-down interest in public and patient involvement and engagement (PPIE). In the United Kingdom health-related sphere, PPIE – both engaging people with research and raising their awareness about it – is considered important and expected by funders such as the NIHR to feature in projects and activities [25, 49, 50]. This interest extends beyond healthcare research; citizen engagement in policy formation is expected in an increasing number of councils [51].

Our respondents clearly identified benefits of working across council departments, particularly in relation to addressing the wider determinants of health. But they also recognized that research culture and what counts, and is valued, as evidence varies across departments within the same local government. This has been recognized elsewhere [20]. Reflective and co-productive spaces need to be considered within the council as well as with academic partners to tackle the wider determinants, and this has messages for within-council research infrastructure such as research interest groups and communities of practice. This realization is also important so local government officers can operate effectively across departments and organizations in complex systems [41].

Our findings also resonate with other more organizationally focused mechanisms identified by Cooke and colleagues [8]: those of “making research core business”, “releasing resources” and “modelling positive behaviours”. Again, our findings may help shape such mechanisms but also highlight nuances to support them to operate in local government. There was an expectation that the council should be research-led, implying research should be seen as core business, but there were some concerns whether this really happened at all levels of the organization. Although this was a clear vision, barriers were identified to making it happen. These included protected time, budget, finance and other resource limitations, competing priorities and the uneven distribution of research skills and academic contacts. These issues have been supported elsewhere in literature [19, 38, 52] and are evident within the wider health system [53–56]. However, respondents in this study offered some solutions. These include focusing on undertaking research that addresses local needs, strong leadership recognizing research as a legitimate activity and incorporating research in systems processes and governance within the authority, for example, including research as part of committee proceedings, job descriptions and appraisals.

Literature supports a system approach to public sector improvement such as RCD [41, 57]. The LARS report identified opportunities for developing research capacity and stimulating research activity through HR systems and processes, including job descriptions, personal development plans and organizational training plans [58]. Small discretionary funding could also be used to support pilot evaluations. It is evident that resourcing RCD is a fundamental element of success [3, 47], and flexible leadership to enable this within organizations is clear in research partnerships and organizations [40]. These are important messages for the future of HDRCs, which have seen enthusiastic uptake across 30 areas [58]. With this or similar substantial and multi-year external funding, there is scope for HDRCs to serve as a springboard to a wider range of opportunities to resource RCD in local authorities.

The “modelling positive behaviour” mechanism [8] was supported by our findings. This included supporting individual research role models and delivering events and activities that champion research activity to demonstrate it can be done. Such events increase pride and promote a research culture. Role models and mentoring were seen as positive aspects of embedding research within local government. However, there was also a view that research existed in specialist pockets. This ideally should be addressed in RCD planning to support wider inclusivity across departments.

The final two mechanisms of “liberating talent” and “learning by doing” [8] were seen as important, but respondents expressed concerns about limited opportunities to enact these. The council has a rich potential for undertaking and delivering research, but this was seen as an untapped resource with little appropriate research training and no awareness of experiential “learning by doing” opportunities such as research secondments, internship and shadowing possibilities for council staff. Some of these are beginning to be developed by the NIHR, but this requires an increase in number and variety.

Several of our findings accord with findings from other LARS projects. These included the fundamental importance of supportive relationships and infrastructure, particular considerations related to the democratic nature of local government, the challenges of limited capacity and resource in attempting to meet heavy and growing demands, the need for local authority leaders to support a strategic approach to research and the value in developing the scope for research, policy and practice to influence one another [59–61]. This suggests that, while some facets of RCD in local authorities may be localized, several appear to be more general, which could inform future practice and policy development intended to address these across local authorities generally.

### Public service: value and leadership skills

Consideration of public value can help local government officers to understand the importance of RCD for themselves, as well as offering a framing narrative in making the case for it to others. This in turn can support the need to make research everyone’s business and releasing resources, both of which were identified as RCD mechanisms by Cooke and colleagues [8] and by interviewees in our study.

Public value [62] is how the public sector can understand the good it generates for society – not just the economic bottom line; public servants achieve this largely by satisfying the wishes of the public and delivering services increasingly efficiently and effectively. While local authority public health research outputs can create public value through better elucidating the needs and desires of the community and what works to address these, the process of RCD can itself cultivate that value where local government research approaches centre community dialogue. The stated desire of our study respondents to approach RCD collaboratively to make a difference for local people aligns with this aspect of creating public value. Indeed, public value itself can be understood as based on and able to strengthen the relationship between communities and local government [63].

The views and values of the public are not only ethically important to incorporate into research – as evident in the RCD literature and echoed by our respondents. Involving the public in and through RCD can link them to key roles designing and delivering effective, well-led and co-produced solutions that grow public value [64]. Indeed, RCD can help local authorities to generate greater public value through increasing the usefulness of interventions, through developing and demonstrating leadership, collaborating and implementing iterative improvement through the dynamics activities for which local authorities are well placed at the nexus of research, policy and practice [64].

Our respondents identified a need for leadership to support and model RCD’s value. Achieving this could support improved public value and RCD. Tammeaid [41] has proposed a range of high-level skills for public leadership that relate to and can underpin RCD on the basis of the mechanisms at the centre of our study. In identifying the need for local government officers to combine capabilities in research orientation, capacity and adaptability, leadership meta-skills offer broad, practicable capabilities that enable success in the complex system working of local government, where officers work at the intersection of the public, other professionals and wider governance.

There is, additionally, some affinity between Cooke and colleagues’ identified RCD mechanisms [8] and these leadership meta-skills for local government officers [41]. For example, “seeing oneself and the actions of one’s own organization as part of the bigger picture” clearly concurs with the theme of “exceeding the sum of the parts” identified from the 2018 synthesis [8] and the findings of our study. The skill of learning to learn can support greater effectiveness of opportunities to learn by doing that supports RCD. Similarly, adopting a systems perspective will support RCD mechanisms not only of “exceeding the sum of the parts” but also “co-producing knowledge”, both of which refer to the need to work beyond organizational or professional boundaries to develop research capacity and transform work and outcomes. Indeed, the necessity of understanding what works for developing research capacity in a whole-systems context is vital in addressing complex public health challenges, and as the English public health system itself changes.

Other meta-leadership skills of importance for local government leaders include “taking a dialogical stance in interaction, harnessing thinking skills, reaching out to practice and adopting an enabling mindset” [65]. These skills link well to themes of importance for RCD identified by our study interviewees and to the underpinning RCD mechanisms [8, 66]. Such skills-behind-the-skills for developing research capacity are indicated not only in identified RCD and leadership actions but also in conditions and capabilities that are found to underpin systems most fit to develop research capacity.

### Strength and limitations

A key strength of this study is that it offers the first examination of affinity between proposed theories or mechanisms in the 2018 synthesis [8] and Cooke [66] in a local government context and may help to support the planning and development of RCD in local government and partnerships addressing the wider determinants within health and care systems.

A limitation of the study is that it is based on data from one local authority and the culture and contexts may vary. However there may be transferable messages, particularly as this is theory driven. These may be limited, however, to local governance bodies in or that are similar to English local authorities, especially in relation to the position and function of public health therein.

## Conclusions

Our findings have implications for RCD in local government. The RCD mechanisms can inform the emerging research infrastructures plans to stimulate joint work, promote synergy and unlock untapped potential. A key message is that such partnerships should focus on local impact for communities and aim to achieve a balance between scientific rigour and actionable, meaningful research. Other messages include developing an architecture or ecosystem that includes space to support ongoing dialogue for co-production across the research–health and care system, and between departments within the authority, to maximize the potential to address the wider determinants. Joint priority setting should be the first step to identify mutual goals. Posts such as embedded researchers, knowledge brokers and role models with flexible and brave leadership have a part to play in such partnerships to reduce tension and build trust. Academics require skills in negotiation and power sharing and understand how research and evidence use works in a political environment. In addition, there may be further value in supporting local government officers to cultivate the meta-skills of leadership as the basis for developing research capability and enabling enactment of identified RCD mechanisms in local authority settings.

Making research a core business within English local authorities should be considered through hardwiring research activity in governance and human resources processes, along with top-down leadership giving permission for bottom-up activity. Importantly, resources should be secured to provide protected time and experiential learning as well as community involvement. Training and access to learning opportunities could promote RCD for individuals, along with a recognition that all parties: academics, local government partners and communities, need to learn together by working together. There is some support in literature for at least a degree of generalizability or transferability from the base case to other related contexts in the case of qualitative research such as this study [67, 68]. Therefore, lessons from this study may offer transferable theoretical insights to inform approaches and underpin practical steps for RCD in other local government settings.

## Data Availability

The semi-structured interview schedule is available from the corresponding author in the case of reasonable requests. The dataset generated from the semi-structured interviews for study is not publicly available to protect privacy of participants; interview materials contain potentially identifiable or sensitive information. This is in accordance with the research governance and ethics policies of the University of Sheffield.
